# Age-Related Differences in Dynamic Interactions Among Default Mode, Frontoparietal Control, and Dorsal Attention Networks during Resting-State and Interference Resolution

**DOI:** 10.3389/fnagi.2017.00152

**Published:** 2017-05-22

**Authors:** Bárbara Avelar-Pereira, Lars Bäckman, Anders Wåhlin, Lars Nyberg, Alireza Salami

**Affiliations:** ^1^Aging Research Center, Karolinska Institutet and Stockholm UniversityStockholm, Sweden; ^2^Umeå Center for Functional Brain Imaging, Umeå UniversityUmeå, Sweden

**Keywords:** brain networks, functional connectivity, interactions, interference resolution, resting-state

## Abstract

Resting-state fMRI (rs-fMRI) can identify large-scale brain networks, including the default mode (DMN), frontoparietal control (FPN) and dorsal attention (DAN) networks. Interactions among these networks are critical for supporting complex cognitive functions, yet the way in which they are modulated across states is not well understood. Moreover, it remains unclear whether these interactions are similarly affected in aging regardless of cognitive state. In this study, we investigated age-related differences in functional interactions among the DMN, FPN and DAN during rest and the Multi-Source Interference task (MSIT). Networks were identified using independent component analysis (ICA), and functional connectivity was measured during rest and task. We found that the FPN was more coupled with the DMN during rest and with the DAN during the MSIT. The degree of FPN-DMN connectivity was lower in older compared to younger adults, whereas no age-related differences were observed in FPN-DAN connectivity in either state. This suggests that dynamic interactions of the FPN are stable across cognitive states. The DMN and DAN were anti correlated and age-sensitive during the MSIT only, indicating variation in a task-dependent manner. Increased levels of anticorrelation from rest to task also predicted successful interference resolution. Additional analyses revealed that the degree of DMN-DAN anticorrelation during the MSIT was associated to resting cerebral blood flow (CBF) within the DMN. This suggests that reduced DMN neural activity during rest underlies an impaired ability to achieve higher levels of anticorrelation during a task. Taken together, our results suggest that only parts of age-related differences in connectivity are uncovered at rest and thus, should be studied in the functional connectome across multiple states for a more comprehensive picture.

## Introduction

Resting-state fMRI (rs-fMRI) measures temporal correlations in spontaneous blood oxygen level-dependent (BOLD) signal fluctuations of discrete brain regions. Coherence in spontaneous activity among brain regions is referred to as functional connectivity, and provides an important measure of information transfer and dynamics in the brain (Shmuel and Leopold, 2008; Damoiseaux and Greicius, 2009). Several studies have shown coherent spontaneous activity within neuroanatomical systems, revealing large-scale functional networks (Damoiseaux et al., 2006; Chen et al., 2008; Biswal et al., 2010; Allen et al., 2011; Power et al., 2011; Yeo et al., 2011; van den Heuvel and Sporns, 2013; Salami et al., 2014a,b). These resting-state networks (RSNs) show strong within-network connectivity and have a particular topological signature. A number of RSNs are now recognized, including the default mode network (DMN; Raichle et al., 2001; Buckner et al., 2008; Andrews-Hanna et al., 2010), the frontoparietal control network (FPN; Vincent et al., 2008; Spreng et al., 2010; Niendam et al., 2012), and the dorsal attention network (DAN; Corbetta and Shulman, 2002; Fox et al., 2006). The latter two are part of the task-positive network (TPN; Fox et al., 2005) and show increased activation during externalized attention-demanding cognitive tasks (Cabeza and Nyberg, 2000; Fox et al., 2005; Dosenbach et al., 2007). In contrast, the DMN has been shown to deactivate during externally focused tasks (Raichle et al., 2001; Buckner et al., 2008), and is instead active during internally focused tasks (Spreng et al., 2010; Spreng and Schacter, 2012) and unconstrained cognition (e.g., mind-wandering; Mason et al., 2007; Buckner et al., 2008; Christoff et al., 2009; Spreng et al., 2009).

The topology of the brain is similar across different cognitive states (Calhoun et al., 2008; Smith et al., 2009; Cole et al., 2014; Krienen et al., 2014). That is, the same functional networks, including TPNs and the DMN, are present during both rest and a number of cognitive tasks. Functional interactions among these networks are critical for integrating resources from distinct brain systems, in order to support complex cognitive functions (Fransson, 2006; Kelly et al., 2008; Hampson et al., 2010; Spreng et al., 2010; Spreng and Schacter, 2012; Elton and Gao, 2014). However, the way in which interactions between TPNs and the DMN are modulated across cognitive states is not well understood. On the one hand, previous studies report moment-to-moment anticorrelations (i.e., negative correlations) between the DMN and some parts of the TPN, particularly the DAN, during both rest (Fox et al., 2005, 2009; Fransson, 2005; Keller et al., 2015) and task (Fornito et al., 2012; Elton and Gao, 2014, 2015). Importantly, the degree of anticorrelation tends to increase from rest to task and is associated with level of cognitive performance (Kelly et al., 2008; Hampson et al., 2010; Rieckmann et al., 2011; De Pisapia et al., 2011). On the other hand, positive functional coupling between the DMN and FPN has also been observed during rest and goal-directed internally focused cognitive tasks (Simons et al., 2008; Spreng et al., 2010; Spreng and Schacter, 2012; Bluhm et al., 2011; Gerlach et al., 2011; Leech et al., 2011; Gao and Lin, 2012; Di and Biswal, 2014). Yet, the extent to which these opposite connectivity trends reflect increases or decreases in connectivity as a function of cognitive demands is not clear. This diverse pattern of functional connectivity may reflect that different parts of the TPN serve different functions across different cognitive states. Hence, their dynamic profile and coupling with the DMN may also change. In support of this view, Spreng et al. (2010) provided a first indication that the FPN facilitates the relation between the DMN and DAN, by coupling its activity with one or the other in support of internally or externally-oriented cognition. As the FPN is anatomically interposed between the DMN and DAN, it is well placed to integrate information from both networks (Vincent et al., 2008). Although this model was initially suggested for two different types of goal-directed tasks (autobiographical vs. visuospatial planning), it could be extended to become a hypothetical model of dynamic changes from rest to task. Thus, the first aim of this study is to investigate how dynamic interactions among the DMN, FPN and DAN differ between rest and an external goal-directed task.

Functional interactions between large-scale networks, particularly between the DMN and DAN/FPN during both rest and task, are altered in aging (Grady et al., 2006, 2010, 2016; Andrews-Hanna et al., 2007; Sambataro et al., 2010; Wu et al., 2011; Chan et al., 2014; Geerligs et al., 2014a,b; Li et al., 2015). However, the underlying cause of these disruptions is still under debate. Some studies have reported that older adults show lower levels of within-network connectivity in the DMN when performing external attention-demanding tasks, which might lead to disruptive interactions between the DMN and TPNs (Lustig et al., 2003; Grady et al., 2006; Persson et al., 2007; Damoiseaux et al., 2008; Sambataro et al., 2010). Others, however, have suggested that age-related alterations in inter-network connectivity are not caused by dysfunction within the DMN itself, but rather reflect lower flexibility of network interactivity and reduced range of network modulation to changing task demands (Spreng and Schacter, 2012). Thus, this would indicate that there are age-related functional connectivity deficiencies in interactions between the DMN and other networks.

Moreover, the way in which these RSNs are affected by aging may not be identical during rest and task. For some networks, functional connectivity can represent a stable characteristic of the brain, whereas for others it can change depending on cognitive state. Previous studies suggest that both stability and variability are important in shaping individual functional connectivity profiles (Cole et al., 2014; Geerligs et al., 2015). Still, it remains unclear whether the degree of functional connectivity between TPNs and the DMN is stable or whether it differs across states. Geerligs et al. (2015), showed that average functional connectivity among several RSNs was highly similar across mental states, whereas age-related differences remained similar for some RSNs, but different for others. This dichotomy could reflect underlying differences in connectivity nature. Still, regardless of the role of the FPN in supporting internal or external goal-directed cognition, results from previous studies show that the level of functional connectivity within these TPNs and between the FPN and DMN is positive (Spreng et al., 2010; Spreng and Schacter, 2012; Elton and Gao, 2015). On the other hand, the DMN and DAN have been consistently reported to be anticorrelated, reflecting the extent to which the DMN is suppressed and the DAN is engaged (Fox et al., 2005, 2009; Elton and Gao, 2014, 2015). Hence, it is possible that age-related alterations in connectivity between the FPN and DMN/DAN behave differently from those involving the DMN and DAN and, subsequently, exhibit distinct stability patterns. The second aim of our study is to investigate this possibility, by exploring whether possible age-related differences in dynamic interactions among the DMN, FPN and DAN are modulated when switching from rest to task. This allows us to discriminate if age-related differences are readily observed during rest, or whether networks need to be engaged in a task for them to be detected.

We used fMRI data from 29 younger and 30 older participants scanned during rest and while performing the Multi-Source Interference task (MSIT, Bush et al., 2003). To complete the MSIT, subjects need to ignore irrelevant information and deal with multiple dimensions of cognitive interference. This type of conflict resolution is known to decline in aging, with older adults being less able to inhibit irrelevant information (Hasher et al., 1991; Stoltzfus et al., 1993; Madden et al., 2004; Gazzaley et al., 2008; Greenwood and Parasuraman, 2012; Salami et al., 2014b). To investigate how dynamic interactions among these networks change from rest to the MSIT, we used independent component analysis (ICA) to identify the three RSNs. First, we hypothesized that the FPN is more coupled to the DMN during rest, and to the DAN during the MSIT. Second, we predicted that the dynamic coupling between the FPN and DMN during rest and between the FPN and DAN during task is less expressed in older adults. Thus, finding age-related differences during both rest and task would indicate that the functional connectivity profile among these networks is stable across states. If these differences were to vary from rest to task, it would rather imply that functional connectivity changes in a state-dependent manner. Third, we expected the DMN and DAN to be negatively correlated during both states, but that the degree of anticorrelation would be greater during the task. In agreement with past work (Hampson et al., 2010; Rieckmann et al., 2011; Hermundstad et al., 2014), we also examined possible associations between DMN-DAN functional connectivity and cognitive performance. Complementary analyses were carried out to investigate whether resting cerebral blood flow (CBF) within the DMN relates to the level of anticorrelation between the DMN and DAN during the MSIT (Riedl et al., 2014). This would clarify whether lower DMN neural activity during rest underlies an impaired ability to achieve higher levels of anticorrelation during a task.

## Materials and Methods

### Participants

Twenty-nine younger (mean age 25.0 ± 3.4 years, range 20–31, 16 women) and 30 older (mean age 68.2 ± 2.6 years, range 65–74, 16 women) adults from Stockholm, Sweden were sampled. All participants were right-handed, native Swedish-speakers, had normal or corrected to normal vision and no history of neurological illness. None of them reported or was diagnosed with cognitive impairment. There were no significant differences in years of education (young: 14.8 ± 2.1; old: 14.4 ± 3.7), the Mini Mental Status Examination (MMSE; Folstein et al., 1975; young: 29.3 ± 0.7; old: 29.0 ± 0.9), depressive symptoms as assessed using the Swedish version of the Geriatric Depression Scale (Brink et al., 1982; Gottfries, 1997; young: 1.4 ± 1.6; old: 1.5 ± 2.5), or in the state scale of the State-Trait Anxiety Inventory (Spielberger et al., 1983; young: 30.5 ± 5.4; old: 27.9 ± 8.0). We used a cutoff of 24 for MMSE (Folstein et al., 1975) and performed additional behavioral analyses, where older participants showed typical patterns, with worse performance in working memory (*p* < 0.001), but better performance in semantic memory (*p* < 0.001), compared with their younger counterparts. All 59 subjects underwent 6 min of rs-fMRI and 57 subjects also completed the MSIT in the scanner. Three persons (two old, one young) were excluded from analysis due to low task performance (3 SD ± mean). Another two older subjects were excluded for not performing the task at all. One young subject was excluded due to technical error. Thus, the effective MSIT sample included 26 younger and 25 older participants. All subjects gave written informed consent. The protocol was approved by the Karolinska Institutet Ethics Committee in accordance with the recommendations of the Declaration of Helsinki.

### Data Acquisition

Brain imaging data were acquired with a 3T fMRI Siemens Magnetom TrioTim scanner at Huddinge Hospital, Stockholm, Sweden, with a 32-channel head coil. Functional data were obtained with a gradient-echo planar imaging (EPI) sequence as follows: TR = 2.5 s, 39 slices (3.0 mm thick), voxel size 3 × 3 × 3 mm, FOV = 230 mm, flip angle = 90°, TE = 40 ms. Four dummy scans were obtained to allow for equilibration of the fMRI signal. Structural high-resolution T1-weighted images (200 slices, 1 mm thickness, FOV = 256 mm, voxel size = 1 × 1 × 1 mm^3^) were collected after the functional images.

**Figure 1:**
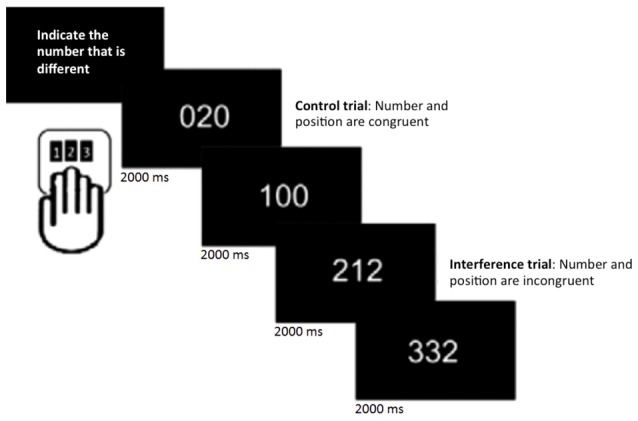
**Multi-Source Interference task (MSIT; Bush et al., 2003).** In each trial, participants were asked to indicate the number that was different by pressing the spatially corresponding key on the button pad.

Participants underwent 6 min of rs-fMRI, during which they were instructed to keep their eyes open and lie still. In addition, they performed the MSIT (Bush et al., 2003), a task that consists of detecting and reporting the number that is different (target) from two other numbers (distracters) presented simultaneously on a screen. It includes 16 blocks of control and interference trials, which alternate during the session. Within each block, 12 stimuli were presented for 2 s each. Participants were given a button box and told that the keys corresponded to numbers 1, 2 and 3, from left to right. They were instructed to indicate the number that was different by pressing the key that spatially corresponded to the target number, regardless of its position on the screen. During control trials, distracters were always zero and the target number corresponded to its position on the button box (e.g., the number 1 always appeared in the leftmost position). In contrast, during interference trials, distracters were 1, 2, or 3 and the target never matched its position on the keyboard. Participants were instructed to respond as accurately and quickly as possible (Figure 1). Stimuli were presented on a computer screen that was seen by participants through a tilted mirror attached to the head coil. E-prime (Psychology Software Tools, Inc., Pittsburgh, PA, USA^1^) was used for presentation of stimuli and responses were made on custom-built MRI-compatible response pads (MAG Design and Engineering, Sunnyvale, CA, USA).

CBF data were acquired using a pseudo-continuous arterial spin labeling (pCASL) sequence with the following settings: TE/TR = 18/3500 ms, 18 slices (6.0 mm thick), FOV = 230 × 230, flip angle = 90°, labeling duration = 1600 ms, post-labeling delay = 1170 ms, matrix size = 64 × 64, inter-slice gap = 0.9 mm, bandwidth = 2790 Hz/pixel, with 70 control/label acquisitions.

### Data Analysis

#### Preprocessing

Functional and structural images were preprocessed using Statistical Parametric Mapping Software (SPM12; Wellcome Department of Imaging Science, Functional Imaging Laboratory, University College London). All functional images were first corrected for differences in slice-time acquisition within each volume using the middle slice as reference. The resulting slice-timing corrected images were rigidly aligned to the first volume to correct for head motion. These images were then despiked with 3dDespike in AFNI^2^, which minimizes the effect of outliers by eliminating spikes in the time-series signal. Despiking is very similar to the scrubbing method proposed by Power et al. (2012), but rather than removing the affected time points, the outliers are replaced with estimates derived from a third-order spline fit. Next, a within-subject rigid registration was conducted in order to align functional and structural images. T1-weighted images were then segmented into gray matter (GM) and white matter (WM), and a group-specific template was created with Diffeomorphic Anatomical Registration using Exponentiated Lie Algebra (DARTEL; Ashburner, 2007). GM and WM images were imported into the DARTEL space using the normalization parameter previously generated during segmentation, followed by resampling to isotropic voxels. A first template was produced as a mean of GM/WM across all subjects. Then a deformation from this template was computed to each of the subject-specific GM/WM images. The inverse of the deformation was applied to the subject-specific GM/WM images. A second template was produced as the mean of the deformed subject-specific GM/WM images. This included six iterative steps of increasingly improved group-specific templates. The realigned fMRI and segmented GM/WM images were then non-linearly normalized to a sample-specific template, affine aligned to the Montreal Neurological Institute (MNI) template, and smoothed using a 6 mm full-width at half-maximum (FWHM) Gaussian filter. For smoothing, we followed the theory of Gaussian random fields, according to which reliable estimates of statistical significance can only be obtained when smoothing kernels have at least twice the voxel size (Worsley and Friston, 1995).

The pCASL postprocessing was based on scripts provided in the ASL toolbox^3^. It included motion correction by rigid body transformation, creation of a mean image, coregistration between the mean image and anatomical T1, realigning the pCASL images to match the mean, spatially smoothing the data (6 mm FWHM Gaussian filter), and calculating a CBF map in ml/100 g/min. The maps were spatially normalized in analogy to the functional scans. Finally, a threshold map was used to calculate subject specific CBF in the DMN. As a control analysis, a similar procedure was undertaken to calculate CBF in the primary visual network.

#### Statistical Analyses

ICA was applied to the resting-state preprocessed images using the group ICA fMRI toolbox (GIFT v2.0a; Calhoun et al., 2001; Allen et al., 2011). ICA is a multivariate data-driven technique that decomposes the fMRI dataset into independent spatial maps and respective time courses. This is done by first reducing the intensity-normalized data from each subject using principal component analysis (PCA), which decreases computational complexity while keeping most of the information. The resulting volumes were then temporally concatenated and PCA was performed again. After these two steps of data reduction, ICA was performed using the Infomax algorithm to identify 21 independent components (ICs), estimated by minimum description length criteria (MDL), on a group level. Finally, a back reconstruction using an improved version of dual regression (GICA3, Erhardt et al., 2011) was carried out, and spatial maps and corresponding time courses were computed for each subject. After visually inspecting all 21 ICs and comparing their topology to those of previous studies, 13 were considered to represent relevant RSNs (Raichle et al., 2001; Damoiseaux et al., 2006; Andrews-Hanna et al., 2007; Smith et al., 2009; Biswal et al., 2010; Allen et al., 2011; Salami et al., 2014b). These networks exhibited spatial overlap with RSNs identified in previous studies (Biswal et al., 2010; Di and Biswal, 2014; Salami et al., 2014b), showed peak activation in the GM, and had little to no overlaps with ICs known to reflect vascular, ventricles, motion and susceptibility artifacts. Out of the 13 relevant networks, we identified and further analyzed the right and left FPN, DMN and the DAN. The inter-network functional connectivity, which reflects the degree of cross talk between two specific networks, was then computed. This was carried out using Fisher’s z-transformed Pearson correlation coefficients between pairs of time courses that were previously detrended, despiked, and filtered using a fifth-order Butterworth low-pass filter (*f* < 0.15). Importantly, given that previous studies have shown that head motion in the scanner can have a strong effect on functional connectivity during rest (Power et al., 2012; Buckner et al., 2013), additional preprocessing steps and control analyses were carried out in order to distinguish noise sources from the signal of interest. Outliers from subjects’ time courses were identified based on the median absolute deviation and replaced with the best estimate using third-order spline fit. Previous work has shown that this method is efficient in reducing the effect of head motion from ICA time courses (Allen et al., 2014; Geerligs et al., 2014a; Salami et al., 2016). As a control analysis, an additional step was carried out where 24-motion parameters using the Friston model (Yan et al., 2013) were regressed out before performing the ICA.

Because we were also interested in investigating the degree of change in functional connectivity during the interference resolution task, a constrained ICA (Calhoun et al., 2005) was applied to the preprocessed MSIT fMRI images, using the templates derived from the resting-state ICA analyses. The inter-network connectivity between the components of interest was also computed. Then, in order to assess whether connectivity between these networks changes from rest to task, a 3 (connectivity: FPN-DMN; FPN-DAN; DMN-DAN) by 2 (state: rest vs. task) repeated-measures analysis of variance (ANOVA) was conducted. The ANOVA was first carried out for the younger group only, to assess how inter-network connectivity changes in a canonical sample composed by healthy young individuals. This was followed by a 3 (connectivity: FPN-DMN; FPN-DAN; DMN-DAN) by 2 (state: rest vs. task) by 2 (group: young vs. old) ANOVA, in order to compare connectivity differences between the two groups. When appropriate, results were followed by *post hoc* *t*-tests (Bonferroni corrected for multiple comparisons).

In addition, we calculated the degree of task-relatedness for each of these networks with the temporal sorting option in GIFT. This method uses a multiple regression fit to each subjects’ ICA time courses. First, regressors modeling both the control and interference conditions were computed using SPM12, by convolving the ideal timing of the events with a canonical hemodynamic response function. Then, these regressors were fit to subjects’ time courses and the average percent signal change was computed. Task-relatedness was measured by analyzing the fit parameters. A network would be task-related if the regressor parameter fit survived a one-sample *t*-test (Calhoun et al., 2008). Finally, to test for an association between functional connectivity and MSIT performance, we computed change-change correlations for inter-network functional connectivity (connectivity during rest—connectivity during task) and MSIT accuracy (accuracy in control condition—accuracy in interference condition). This analysis was carried out for the FPN-DMN, FPN-DAN and DMN-DAN. We also tested whether resting CBF in the DMN was associated with the level of DMN-DAN anticorrelation during task performance.

## Results

### Cognitive Performance

A 2 (condition: control vs. interference) by 2 (group: young vs. older) ANOVA was conducted on the accuracy data. The analysis showed a main effect of condition (*F*_(1,104)_ = 16.146, *p* < 0.0001), a main effect of age (*F*_(1,104)_ = 4.397, *p* = 0.038) and a significant age × condition interaction (*F*_(1,104)_ = 4.239, *p* < 0.05). Older subjects’ were less accurate during interference than during the control condition (*p* = 0.002), but this effect was only at trend level in younger adults (*p* = 0.08). The older group was also less accurate compared to the young during interference (*p* < 0.05), but not during control (*p* = 0.9). A similar ANOVA was run for latency for correct trials, showing significant main effects of condition (*F*_(1,108)_ = 108.912, *p* < 0.0001) and age (*F*_(1,108)_ = 21.830, *p* < 0.0001), but no interaction (*F* < 1). Reaction times were longer for interference compared to control trials for both groups (*p* < 0.0001), and longer for older adults than for the young in both conditions (*p* < 0.05; for more details see Salami et al., 2014b).

### Mapping Resting-State Networks

**Figure 2:**
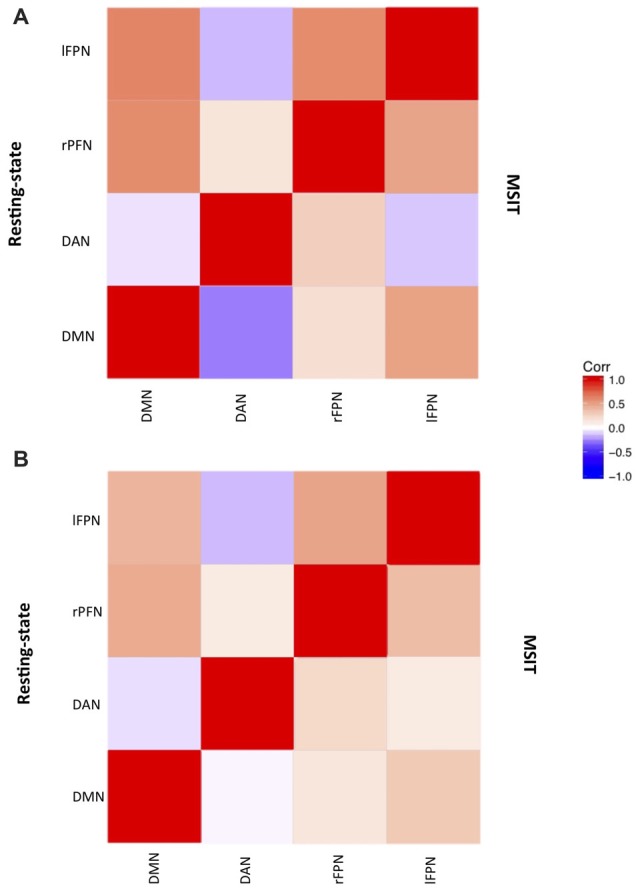
**Networks correlation matrix during resting-state (upper-case diagonal) and interference resolution (lower-case diagonal) for (A)** young and **(B)** old.

ICA estimated a total of 21 components, 13 of which represented RSNs. We identified the DMN, bilateral FPN and DAN (Figure 2), by comparing the topology of all ICA components with those of previous studies (Spreng et al., 2010; Di and Biswal, 2014; Salami et al., 2014b).

**Figure 3:**
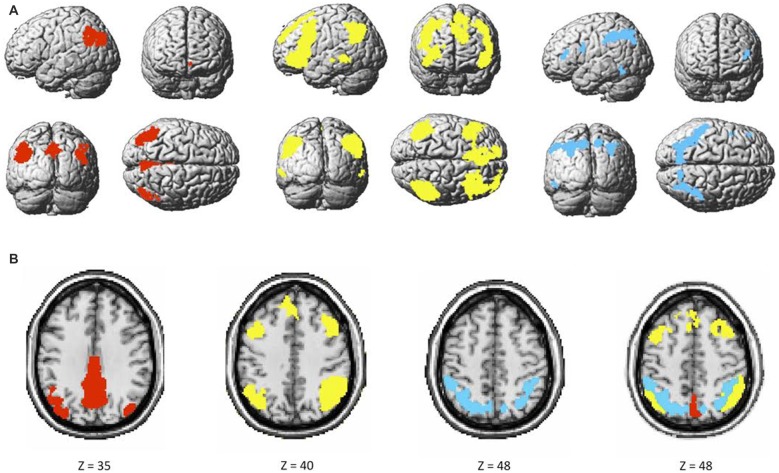
**(A)** Maps of the default mode (DMN; red), bilateral frontoparietal control (FPN; yellow), and dorsal attention (DAN; blue), and** (B)** overlap among the three networks.

The two components identified as the DMN were averaged, and consisted of brain regions traditionally known to be part of this network such as the ventral medial prefrontal cortex (vmPFC), inferior parietal lobule (IPL) and posterior cingulate cortex (PCC). The FPN included the rostrolateral prefrontal cortex (rlPFC), anterior extent of the inferior parietal lobule (aIPL), and middle frontal gyrus (MFG). Finally, the DAN consisted, among others, of the dorsolateral prefrontal cortex (dlPFC) and superior parietal lobule (SPL). These networks had little to no overlap with known artifacts or with each other (Figure 3).

### Inter-Network Connectivity in the Younger Group

We first investigated whether the degree of functional connectivity among the DMN, FPN (averaged across the two hemispheres), and DAN changed from rest to the MSIT in the group of younger subjects. This also served as a check to assess whether functional connectivity levels fell within the expected range. The ANOVA revealed a main effect of connectivity (*F*_(2,54)_ = 305.76, *p* < 0.0001), a main effect of state (*F*_(1,27)_ = 23.02, *p* < 0.0001) and a connectivity × state interaction (*F*_(2,54)_ = 44.67, *p* < 0.0001). Pairwise comparisons showed that FPN-DMN connectivity was higher (*t*_(27)_ = 7.34, *p* < 0.0001) at rest compared to task, whereas the opposite pattern was seen for the FPN and DAN, where functional connectivity was higher (*t*_(27)_ = −2.57, *p* = 0.016) during the MSIT as compared to rest. Moreover, the level of DMN-DAN anticorrelation also increased (*t*_(27)_ = 5.13, *p* < 0.0001) from rest to task.

**Figure 4:**
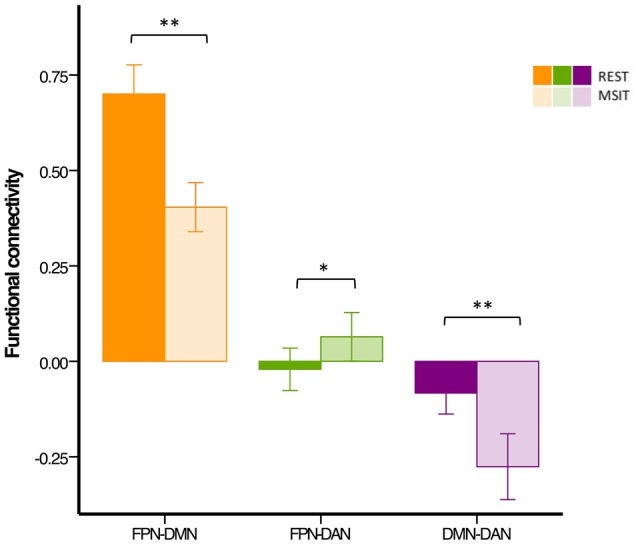
**Functional connectivity among the DMN, FPN and DAN during rest and the MSIT for the younger group.** Connectivity between the FPN and DMN significantly decreased from rest to task, whereas the opposite trend can be seen for coupling between the FPN and DAN. The DMN and DAN were significantly more anticorrelated during task performance than during rest. *p < 0.05; **p < 0.001.

In summary, younger adults had lower FPN-DMN and higher FPN-DAN connectivity during the MSIT, as compared to rest. They also showed a task-related increase in the level of DMN-DAN anticorrelation. However, the degree of correlation between the FPN and DAN was lower (*r* < 0.1) than expected during both states, when compared to the other networks or results found in previous studies (Spreng et al., 2010; Spreng and Schacter, 2012; Figure 4).

We further examined possible reasons for low FPN-DAN connectivity. The magnitude of the correlation between the FPN and DAN was significantly different from zero, but quite low when compared to other networks. Thus, we hypothesized that the FPN—the network responsible for coupling itself with the DMN or DAN according to task demands—could be differentially modulated (i.e., engaged or disengaged) between the right and left hemisphere given the nature of the task.

To investigate this, a multiple regression fit with control and interference conditions as regressors was carried out on subjects’ ICA time courses. We found that the right FPN (rFPN) was strongly and positively related to both control and interference, whereas the left FPN (lFPN) was not significantly associated with any of the two conditions (Table 1 for results across groups). The DAN and DMN were positively and negatively associated to the task, respectively. Finally, the rFPN and DAN were the most task-related networks in our study.

**Table 1:** **Network task relatedness across both age groups**.

	Interference		Control	
	*t*-value	*p*-value	*t*-value	*p*-value
Default mode network (component 1)	−9.748	1.145e-13	−5.834	2.821e-07
Default mode network (component 2)	−6.151	8.596-08	−5.779	3.466e-07
Right frontoparietal network	−7.679	2.614e-10	−8.457	1.374e-11
Left frontoparietal network	−1.528	0.132	−0.668	0.507
Dorsal attention network	11.175	6.999e-16	3.411	0.001

**Figure 5:**
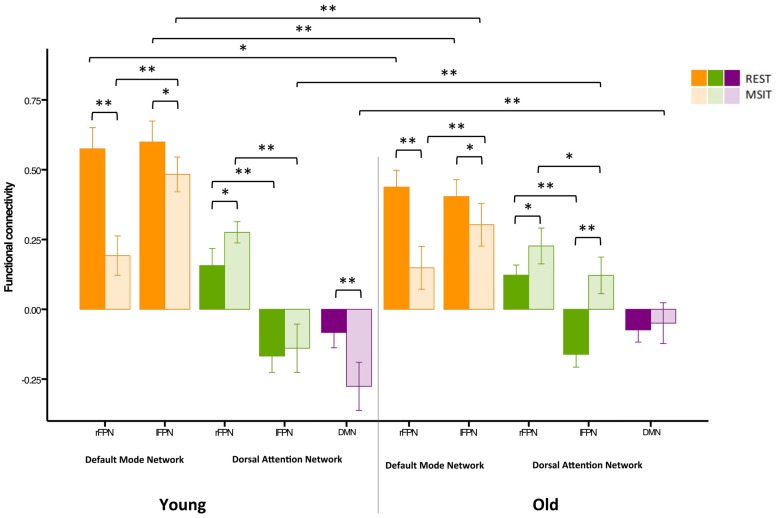
**Functional connectivity among the DMN, FPN and DAN during rest and the MSIT for younger and older adults.** The young had higher FPN-DMN connectivity than the old in both conditions. However, both groups showed a decrease in inter-network functional connectivity during the MSIT when compared to rest. Both groups also showed an increase in right FPN (rFPN)-DAN connectivity during the MSIT compared to rest, but there were no differences between young and old. Although there were also no differences between the groups in lFPN-DAN connectivity during rest, the old showed increased connectivity during the MSIT, whereas the young group retained similar levels of (negative) connectivity. DMN-DAN connectivity levels did not significantly differ between the age groups at rest, but the young showed increased negative connectivity during the MSIT, whereas the old did not. *p < 0.05; **p < 0.001.

Results from the task-relatedness analysis are indicative of a lateralized effect regarding FPN connectivity during the MSIT. Hence, rather than analyzing connectivity with the averaged bilateral FPN, we repeated the analyses for the rFPN and lFPN separately (Figure 5, left panel). When including the rFPN, the ANOVA showed a main effect of connectivity (*F*_(2,54)_ = 160.89, *p* < 0.0001), a main effect of state (*F*_(1,27)_ = 38.27, *p* < 0.0001) and a connectivity × state interaction (*F*_(2,54)_ = 58.62, *p* < 0.0001). In line with the initial results, these data also indicate that rFPN-DMN connectivity was higher (*t*_(27)_ = 10.25, *p* < 0.0001) during rest compared to the MSIT, whereas connectivity between the rFPN and DAN was higher during task (*t*_(27)_ = −3.29, *p* = 0.003) compared to rest. When the model included the lFPN instead, results also showed a main effect of connectivity (*F*_(2,54)_ = 290.25, *p* < 0.0001), a main effect of state (*F*_(1,27)_ = 10.00, *p* = 0.004), and a connectivity × state interaction (*F*_(2,54)_ = 10.94, *p* < 0.0001). Connectivity between the lFPN and DMN was again higher (*t*_(27)_ = 2.53, *p* = 0.018) during rest than during task. However this was not the case for connectivity between the lFPN and DAN, which was not different between the two states (*t*_(27)_ = −0.71, *p* = 0.483).

In sum, similar effects to our previous analysis were found regarding rFPN/lFPN connectivity with the DMN, such that connectivity between the networks decreased from rest to task. Likewise, connectivity between the rFPN and DAN was also consistent with the initial results, showing an increase from rest to task. However, connectivity between the lFPN and DAN remained identical in both states. Thus, in order to facilitate interpretation in the following analyses, we averaged right and left FPN connectivity concerning the DMN. Because connectivity between the FPN and DAN showed distinct unilateral patterns, this could not be done for the DAN.

### Inter-Network Connectivity in the Younger vs. Older Group

Our second aim was to investigate whether functional connectivity changes among the DMN, FPN and DAN were differentially expressed in young and older adults. Hence, a 3 (connectivity) by 2 (state) by 2 (group) ANOVA was conducted using the right lateralized and task-related rFPN-DAN connectivity. This revealed a main effect of connectivity (*F*_(2,110)_ = 382.96, *p* < 0.0001), a main effect of state (*F*_(1,55)_ = 14.91, *p* < 0.0001), but not of group (*F*_(1,55)_ = 1.90, *p* = 0.174), as well as a connectivity × group interaction (*F*_(2,110)_ = 21.20, *p* < 0.0001), a state × group interaction (*F*_(1,55)_ = 7.57, *p* = 0.008), a connectivity × state interaction (*F*_(2,110)_ = 54.89, *p* < 0.0001) and a connectivity × state × group interaction (*F*_(2,110)_ = 6.74, *p* = 0.002). Specifically, FPN-DMN connectivity was higher during rest than during the MSIT for both the young (*t*_(27)_ = 7.34, *p* < 0.0001) and the old (*t*_(28)_ = 4.43, *p* < 0.0001). The younger group had higher connectivity levels during both rest (*t*_(57)_ = −4.15, *p* < 0.0001) and task (*t*_(55)_ = −0.247, *p* = 0.017) when compared to the older group. In summary, the older group showed significantly lower connectivity during rest and the MSIT when compared to the young, but exhibited the same pattern of results by decreasing the degree of FPN-DMN connectivity during task performance.

Connectivity between the two task-related networks, the rFPN and DAN, was higher during MSIT as compared to rest for both young (*t*_(27)_ = −0.33, *p* = 0.003) and old (*t*_(28)_ = −3.12, *p* = 0.004). Moreover, younger subjects had slightly higher rFPN-DAN connectivity compared to the old, but this difference did not approach conventional significance in either state (rest: *t*_(45.555)_ = −0.99, *p* = 0.329; MSIT: *t*_(45.220)_ = −1.34, *p* = 0.186). Hence, both young and old showed higher connectivity between the rFPN and DAN during the MSIT than during rest, but there were no age-related differences in either state.

Despite not being task-related, pairwise *t*-tests were carried out in order to compare lFPN and DAN connectivity. During rest, the younger and older groups’ connectivity was not different (*t*_(57)_ = 0.16, *p* = 0.872), but during the MSIT, the old showed significantly higher connectivity (*t*_(55)_ = 4.95, *p* < 0.0001), compared to the young. Whereas the young group showed no significant difference in lFPN-DAN connectivity between rest and task (*t*_(27)_ = −0.71, *p* = 0.483), the old group showed an increase in functional connectivity during the MSIT (*t*_(28)_ = −7.42, *p* < 0.0001). This indicates that connectivity between the lFPN and DAN remained the same during both states for the young, but increased during the task for the old.

Finally, the level of DMN-DAN anticorrelation indicated that the younger and older groups’ connectivity was not significantly different during rest (*t*_(57)_ = −0.14, *p* = 0.888), but that the young had higher negative DMN-DAN connectivity compared to the old during the MSIT (*t*_(55)_ = 4.56, *p* < 0.0001). From a different angle, connectivity levels did not differ between the two states in the older group (*t*_(28)_ = −1.48 *p* = 0.151), whereas the degree of anticorrelation increased in the young during the MSIT (*t*_(27)_ = 5.13, *p* < 0.0001). As such, the young showed greater task-related modulation in DMN-DAN anticorrelation compared to the old (Figure 5).

### Correlations with MSIT Performance and Perfusion

**Figure 6:**
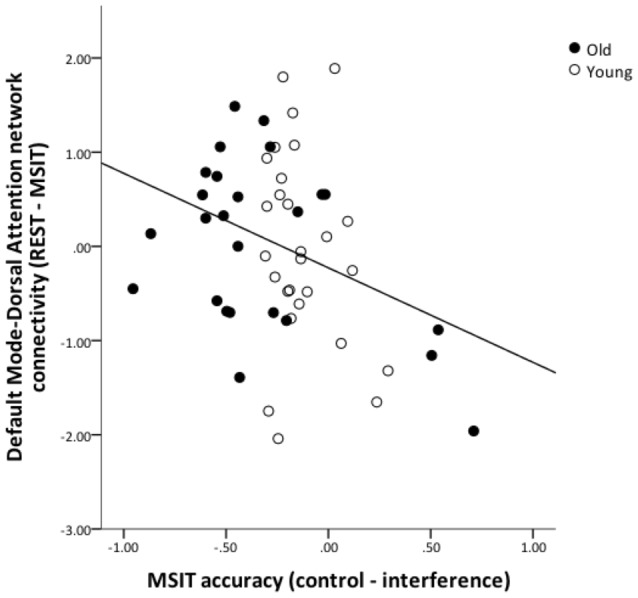
**The relation between DMN and DAN coupling and MSIT accuracy.** The graph shows the residuals of the change-change age-partialed correlation between DMN-DAN connectivity (rest—task) and accuracy during the MSIT (correct responses for control items—correct responses for interference items).

To test for an association between functional connectivity and MSIT performance, we computed change-change correlations for inter-network functional connectivity (connectivity during rest—connectivity during task) and MSIT accuracy (accuracy in control condition—accuracy in interference condition) for all connectivity pairs (FPN-DMN, FPN-DMN and DMN-DAN). Due to the relatively small sample size, these analyses were carried out across all subjects, while controlling for age. The increases in DMN-DAN anticorrelation were significantly correlated with accuracy performance (*r* = −0.339, *p* = 0.015), indicating that better interference resolution was associated with a greater increase in DMN-DAN anticorrelation from rest to MSIT (Figure 6). No other associations were found between inter-network connectivity and performance (*p* > 0.05).

Finally, we investigated whether the degree of anticorrelation between the DMN and DAN during task was related to resting CBF in the DMN. The results showed that higher resting CBF in the DMN was associated with a stronger DMN-DAN anticorrelation during the MSIT (*r* = −0.30, *p* = 0.047, after adjusting for age, DMN gray-matter volume, and CBF in the visual cortex). The old had significantly lower global gray-matter CBF than the young (41 ± 9 vs. 57 ± 12 ml/100 g/min, *p* < 10^−7^). This indicates that high absolute DMN activity during rest contributes to the ability to increase the level of DMN-DAN anticorrelation during a task (Riedl et al., 2014).

### Motion and Age-Related Differences in Connectivity

**Figure 7:**
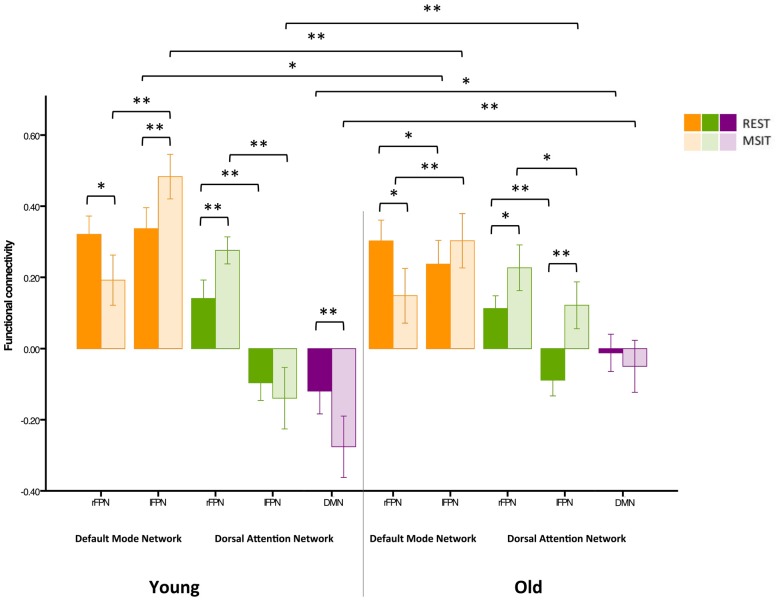
**Control analysis on functional connectivity levels among the DMN, FPN and DAN during rest and the MSIT for younger and older adults.** Connectivity between the rFPN and DMN decreased for both groups from rest to task. The younger also had higher connectivity between the lFPN and DMN during the MSIT when compared to rest, whereas the old showed no connectivity differences between states. Importantly, there were no differences in rFPN-DMN connectivity between groups during both states, whereas connectivity between the lFPN and DMN was higher for young during both rest and task. Hence, despite exhibiting seemingly opposite effects, the same trend is still observed—an age-related difference that is present during one state is also present during the other and vice-versa. In line with our previous findings, both groups showed an increase in rFPN-DAN connectivity during the MSIT compared to rest, and there were no differences between groups. Young also showed negative connectivity between the lFPN and DAN, with no significant difference between rest and task, whereas the old group’s connectivity increased during the MSIT. This is also consistent with our previous results, where young and old similarly increased rFPN-DAN connectivity during the MSIT, but there were no age-related differences in either state. The same trends are also present for the lFPN and DAN, indicating that the young did not change their connectivity levels from one state to the other, whereas the older group did. Finally, both groups had negative connectivity between the DMN and DAN, but the young showed increased anticorrelation during the task while older subjects did not. Importantly, the correlation between DMN-DAN connectivity changes and MSIT performance remained after regressing out motion (*r* = −0.408, *p* = 0.003). *p < 0.05; **p < 0.001.

Previous studies have indicated that head motion can create confounds in functional connectivity (Power et al., 2012; Buckner et al., 2013). As such, we have already motion-corrected subjects’ ICA time courses, which were then used to compute inter-network connectivity. However, in order to further investigate whether our results were confounded by motion, we carried out an additional control analysis where 24-motion parameters using the Friston model were regressed out, before the ICA—as opposed to motion correction on the ICA time courses. This analysis revealed very similar results to our previous findings (see Figure 7).

## Discussion

The primary aim of this study was to investigate state-dependent changes in dynamic interaction patterns of three large-scale brain networks in younger and older adults. By comparing inter-network functional connectivity during rest and the MSIT, we demonstrated that interactions within TPNs (i.e., FPN and DAN) and between TPNs and the DMN differ between rest and task in both young and old. Specifically, the FPN was more coupled to the DMN during rest, and more coupled to the DAN during the MSIT in both age groups. Past research has shown increased functional connectivity between the FPN and DMN during autobiographical planning and between the FPN and DAN during visuospatial planning (Spreng et al., 2010; Spreng and Schacter, 2012). Our results further demonstrate that the interposition of the FPN between the DMN and DAN represents a robust effect, and support a switching role for the FPN by dynamically interacting with one or the other depending on task demands. Past work using graph theory has provided more direct evidence for this switching function, by showing that the FPN includes brain regions that flexibly and rapidly update their connectivity in a task-dependent manner, but also that its connectivity pattern shifts more than that of other networks across a variety of tasks (Cole et al., 2013). Moreover, a study by Spreng et al. (2013) identified FPN nodes that exhibit distinct preferred connectivity with the DMN, DAN, or both. These nodes changed their network affiliation and showed realignment from rest to task, which suggests a more flexible connectivity profile.

Our second aim was to investigate age-related differences among the DMN, FPN and DAN. We found that older adults had lower FPN-DMN functional connectivity during both rest and the MSIT, but still exhibited greater FPN-DMN connectivity at rest compared to task. A similar trend was observed for interactions between the rFPN and DAN, with older adults showing numerically lower connectivity values for both states compared to the young, but these did not reach conventional significance. Older adults also had greater rFPN-DAN connectivity during the MSIT compared to rest. These findings suggest that, similarly to what was observed in younger adults, the FPN serves as a switch to actively engage other networks and facilitate cognition in older adults. This pattern is in line with previous studies indicating that normal aging is accompanied by a lower degree of flexible network interactivity (Spreng and Schacter, 2012; Chan et al., 2014; Grady et al., 2016).

Additional analyses revealed that the DMN and DAN were anticorrelated during both states for young and old, but that the degree of anticorrelation increased from rest to the MSIT in the young only. Several studies indicate that these two networks subserve different cognitive functions, with the DMN being more engaged in internally-directed attention (Simons et al., 2008; Spreng et al., 2010; Spreng and Schacter, 2012; Bluhm et al., 2011; Gerlach et al., 2011; Leech et al., 2011; Gao and Lin, 2012; Di and Biswal, 2014), and the DAN being more engaged in externally-directed attention (Corbetta and Shulman, 2002; Fox et al., 2005; Fransson, 2005; Corbetta et al., 2008; Fox et al., 2009; Keller et al., 2015). Our results corroborate that DMN-DAN anticorrelation transcends cognitive states. We also found that changes in DMN-DAN connectivity levels between rest and task were associated with MSIT accuracy, further supporting its behavioral relevance. Changes in connectivity between cognitive states have been associated with performance in the past (Hermundstad et al., 2014); however studies investigating the relationship between DMN-DAN anticorrelation and task performance have mostly focused on memory (Hampson et al., 2010; Rieckmann et al., 2011). Our results extend these patterns to the domain of interference resolution. Furthemore, the degree of anticorrelation during the MSIT was associated with CBF in the DMN during rest. This suggests that DMN activity at rest is a helpful indicator of the degree of change in DMN-DAN connectivity.

In contrast with previous studies, there were no age-related differences in the level of anticorrelation between the DMN and DAN at rest (e.g., Wu et al., 2011; Keller et al., 2015). However, most of these studies report differences in connectivity between the DAN and the anterior, but not posterior, part of the DMN. Likewise, some previous observations regarding age-related differences in anticorrelation levels should be interpreted with caution due to the use of global signal regression, a method that mitigates physiological noise in resting-state but has been shown to mathematically generate anticorrelations (Murphy et al., 2009). A recent study by Spreng et al. (2016) has similarly demonstrated that DMN-DAN anticorrelation is reduced in older adults during both rest and task, which supports the notion that altered network dynamics is a central feature of brain aging. Our results also showed increased anticorrelation from rest to MSIT in the young only. This finding is in agreement with past work showing that both DMN deactivation and increased anticorrelation levels are related to elevated task demands (Kelly et al., 2008; Hampson et al., 2010). The lack of task modulation in the degree of DMN-DAN anticorrelation in the older group is also in line with the view that aging is accompanied by impaired flexible network interactivity (Spreng et al., 2016).

Our task-relatedness analysis indicated that only the rFPN was involved in MSIT performance. Connectivity between the lFPN and DAN was not significantly different between the age groups at rest, although the older group showed increased lFPN-DAN connectivity during the task and the younger group did not. This finding is in line with a previous study where older adults showed increased activity in the left prefrontal cortex (PFC) and parietal regions during sustained visual attention (Cabeza et al., 2004), a necessary component when performing the MSIT. Furthermore, some PFC regions that show lateralized activation in young adults also show more bilateral activity in older adults (Bäckman et al., 1997; Cabeza, 2002; Cabeza et al., 2002). Likewise, previous research has found that older subjects show increased bilateral functional connectivity in the PFC during task performance (Grady et al., 2010; Rieckmann et al., 2011). Thus, older adults might need increased bilateral connectivity in order to adequately perform the task, although this pattern could also reflect less selective recruitment of brain networks, consistent with the concept of dedifferentiation in cognitive aging (Li and Lindenberger, 1991).

The impact of task demands on functional connectivity was also investigated using task-relatedness analyses, where we found that the level of modulation in the three networks differed. Whereas rFPN/DAN and DMN were positively and negatively task-related, the lFPN was task-unrelated. Previous research has indicated that the MSIT activates the cingulo-frontal-parietal attention network bilaterally and does not show a lateralized pattern of activation (Bush et al., 2003; Bush and Shin, 2006) like the one found in our study. The current finding of lateralization within the FPN contrasts with past work, but it should be noted that previous MSIT studies examined activation, and not connectivity. Moreover, sustained attention processes have been associated with the right PFC, which provides a plausible explanation for the lateralization effect found in the current study (Pardo et al., 1991; Lewin et al., 1996; Cabeza and Nyberg, 2000). Finally, whereas the rFPN showed little modulation between conditions (i.e., interference vs. control), the DAN was more involved during interference than control trials, indicating a higher degree of modulation and increased recruitment when task difficulty increased.

We found that age-related differences in functional connectivity were not always consistent across cognitive states, and might be dependent on task demands (Di et al., 2013; Gonzalez-Castillo et al., 2015; but see Tavor et al., 2016). There is a strong body of evidence relating aging to disruptions in network interactions, but it remains unclear whether age-related differences in RSNs are consistently found across cognitive states. Previous research has found that age-related differences in inter-network dynamics are not static across cognitive states (Geerligs et al., 2015). However, this contrasts against other studies showing that individual differences in cognitive tasks may be a stable trait marker (Tavor et al., 2016), or that age differences may be more readily observed when there are no external demands on cognitive processing (Grady et al., 2016). This work provides novel insights as to whether age-related differences in network interactions can be easily identified during rest, or whether networks should be momentarily engaged in a cognitively demanding task to elicit patterns of age-related differences. In our study, age-related differences in connectivity were stable between rest and task for FPN-DMN and FPN-DAN interactions (Tavor et al., 2016). This finding, along with well-known age differences in cognitive control (Buckner, 2004; de Frias et al., 2009), suggests that the dynamic functional connectivity of the FPN with other large-scale networks remains similar across cognitive states and can be readily observed during resting-state. In contrast, DMN-DAN connectivity showed a distinct pattern in the two groups. Although young people had increased negative connectivity during the MSIT, older people had similar connectivity levels in both rest and task. This indicates that age differences in the interactions between the two networks function in a state-dependent manner. Previous studies suggest that the DMN exhibits a general dynamic reorganization of its functional connectivity pattern in a task-specific manner (Gao et al., 2013; Elton and Gao, 2015). According to this view, we would expect that the degree of DMN-DAN anticorrelation would vary across cognitive states.

Despite alterations in the degree of modulation, functional connectivity between the FPN and DMN/DAN remained positive and was stable. In contrast, connectivity between the DMN and DAN was negative and varied between rest and the MSIT. These opposite trends could indicate that DMN suppression is highly state-dependent, whereas positive connectivity between other networks, particularly those involving the FPN, has a more stable pattern across states. Moreover, if we wish to argue that cognitive demands are responsible for connectivity differences in young and old from rest to the MSIT, we would expect similar effects when comparing two tasks differing in cognitive load (Grier et al., 2003; Caggiano and Parasuraman, 2004). This needs to be further investigated across different cognitive domains and using different measures of individual differences.

A limitation of this work is that we cannot address all potentially relevant mechanisms by which changes in cerebrovascular physiology in aging (e.g., changes in neurovascular coupling) could impact connectivity measures. There are many factors that can potentially confound group differences in fMRI studies. Given its nature, the BOLD signal is affected by elements that are unrelated to neural activity, such as changes in cerebrovascular reactivity (CVR), CBF and cerebral blood volume (CBV). Because aging is associated with cerebrovascular physiological changes, controlling for such differences is of particular importance when comparing age groups. However, we did not collect information regarding subjects’ vascular profile or compliance with the resting-state protocol. We took this issue into account, by controlling for resting-state fluctuation amplitude (RSFA) in additional analyses that are not reported here. RSFA is a measure that gives comparable results to CO_2_ challenges and breath-hold (BH) tasks (Liu et al., 2013) and is capable of capturing differences between younger and older participants (Kannurpatti et al., 2011). As expected, older subjects had significantly lower RSFA and therefore reduced CVR. However, the basic pattern of age-related differences remained unaltered. This indicates that neurovascular factors are not driving the main pattern in our findings, although we cannot rule out the possibility that these factors are, at least partly, responsible for our results. Our findings could also be biased by scan length, because the resting-state and task sessions had different durations (145 vs. 180 volumes). Still, this was not the case, as we ran the same analyses using only the first 145 task volumes, and results were identical.

Finally, previous work using the same pool of subjects has shown that older persons have marked GM reductions in several brain regions, particularly in anterior parts of the brain (Salami et al., 2014b). There is also evidence that age-related differences in GM and WM affect the brain’s ability to engage and coordinate large-scale functional networks, including the DMN and FPN (Greicius et al., 2009; Horn et al., 2014; Marstaller et al., 2015). Thus, it was expected that GM differences would account for part of the results found in the present study. After controlling for the effects of GM volume on functional connectivity, the overall pattern of age-related differences was identical to the one found before controlling for atrophy, with only FPN-DMN connectivity showing a similar trend but not reaching statistical significance. Indeed, if functional connectivity is a measure of brain integrity, then structural brain changes should account for age-related changes in interactions among large-scale networks (Marstaller et al., 2015).

In summary, our results provide three main findings. First, our analyses of inter-network connectivity support a model in which the FPN dynamically interacts with the DMN or DAN depending on cognitive state in both younger and older adults. Second, the degree of FPN-DMN connectivity during both rest and the MSIT was lower in older compared to younger adults, whereas no age-related difference was observed in FPN-DAN connectivity in either state. These data suggest that dynamic interactions of the FPN are stable across cognitive states. Third, the DMN and DAN were anticorrelated, and the degree of anticorrelation was age-sensitive only during the MSIT (and predictive of task performance), suggesting that it varies in a state-dependent manner. In addition, low DMN-DAN anticorrelation during task was related to low resting metabolism in the DMN, providing further characterization of the physiological underpinnings of these interactions.

## Author Contributions

BA-P contributed to writing the manuscript, as well as analyzing and interpreting the data. AS contributed to writing the manuscript and assisted in analyzing the results. LB and LN supervised the project and contributed to scientific discussions and manuscript writing. AW analyzed and helped interpret the CBF-related data. All authors critically reviewed the content and approved the final version for publication.

## Conflict of Interest Statement

The authors declare that the research was conducted in the absence of any commercial or financial relationships that could be construed as a potential conflict of interest.

## Footnotes

^1^www.pstnet.com/eprime

^2^http://afni.nimh.nih.gov/pub/dist/doc/program_help/3dDespike.html

^3^https://cfn.upenn.edu/~zewang/ASLtbx.php
